# Trends in 340B Drug Pricing Program Contract Growth Among Retail Pharmacies From 2009 to 2022

**DOI:** 10.1001/jamahealthforum.2023.2139

**Published:** 2023-08-04

**Authors:** Sayeh Nikpay, Claire C. McGlave, John P. Bruno, Haishan Yang, Elizabeth Watts

**Affiliations:** 1 Division of Health Policy and Management, University of Minnesota School of Public Health, Minneapolis; 2 Department of Economics, University of Minnesota College of Liberal Arts, Minneapolis

## Abstract

This cross-sectional study assesses the increases and decreases over time in the number of pharmacy contracts, distance from contracting pharmacies, and proportion of pharmacy contracts with safety-net practices in the US.

## Introduction

The 340B Drug Pricing Program allows certain US hospitals and federal grantees to purchase discounted prescription drugs and bill insurers to generate revenue to care for safety-net–reliant patients. As 340B has grown to account for 7.2% of gross drug spending, participants or covered entities (CEs) increasingly contract with pharmacies to dispense discounted drugs.^1,2^ This mutually beneficial relationship increases revenue for the CEs, and pharmacies commonly receive dispensing fees. Since the Health Resources and Services Administration (HRSA) lifted the limit of 1 contract pharmacy per CE in 2010, 33% of retail pharmacies have at least 1 contract and 76% of US counties contain at least 1 contract pharmacy.^3,4^ However, pharmacies’ involvement with 340B may vary by number of contracts, distance from contracting CEs, and proportion of contracts with core safety-net CEs. We developed measures to describe alternative forms of contract pharmacy growth beyond simple participation.

## Methods

We linked HRSA’s contracts database to retail pharmacies from the National Council for Prescription Drug Programs dataQ database to create an annual, pharmacy-level data set for 2009 to 2022. The University of Minnesota Institutional Review Board deemed this cross-sectional study exempt from review because it was not human participant research. We followed the STROBE reporting guideline.

We examined distributions of depth, spread, and safety-net composition over time. Depth was the number of contracts with unique CEs per pharmacy. Spread was the maximum straight-line distance in miles between the zip code population centroid of the pharmacy and its CEs. Safety-net composition was the proportion of contracts with federal grantees and hospitals meeting the definition of essential community hospitals by Medicaid and Children’s Health Insurance Program Payment and Access Commission.^5^ After eliminating pharmacy-years that could not be linked to dataQ using 340B contract information (n = 4290) and nonretail pharmacy-years (n = 29 728), we included 893 952 pharmacy-years.

We used χ^2^ tests to assess differences in distributions of these measures between 2009 and 2022, and we estimated pairwise correlations between measures in 2022. Two-sided *P* < .05 indicated statistical significance. Data analysis was performed with Stata 17 (StataCorp LLC).

## Results

**Figure 1.**
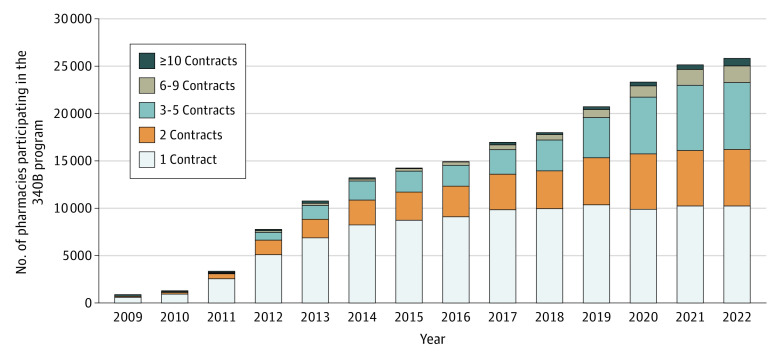
Distribution of Contract Pharmacy Depth Among Retail Pharmacies From 2009 to 2022 Depth is the number of unique, active, 340B contracts with covered entities held by each pharmacy. Retail status was defined using the National Council for Prescription Drug Programs primary type code identifier.

The number of retail pharmacies participating in 340B increased from 789 in 2009 to 25 775 in 2022 or from 1.3% to 40.9% of all retail pharmacies, respectively (Figure 1). Depth increased over time. In 2009, 81% of contract pharmacies had only 1 contract, and by 2022, 40% had 1, 23% had 2, 27% had 3 to 5, 7% had 6 to 9, and 3% had 10 or more (*P* < .001).

**Figure 2.**
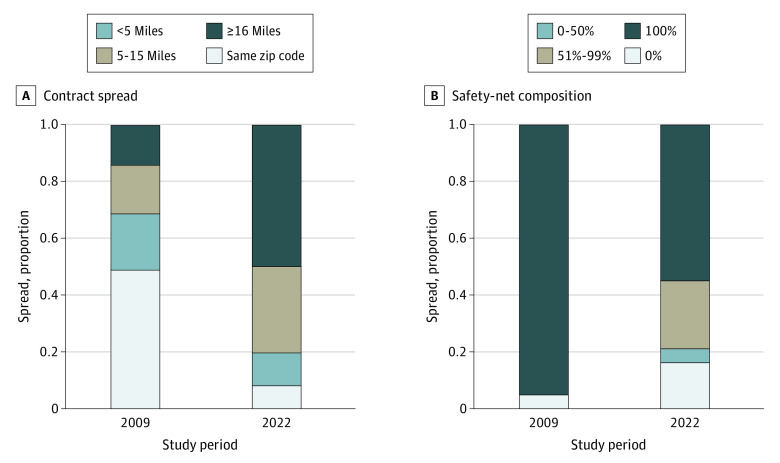
Distribution of Contract Spread and Safety-Net Composition Among Retail Pharmacies From 2009 to 2022 Spread is the distance between the zip code centroid of the pharmacy and the contracting covered entity. Safety-net composition is the share of a pharmacy’s contracts with federal grantees or hospitals that meet the Medicaid and Children’s Health Insurance Program Payment and Access Commission’s definition of essential community hospitals.

Spread increased over time (Figure 2). In 2009, the farthest CE was within the same zip code for 48% of pharmacies, less than 5 miles for 19%, 5 to 15 miles for 17%, and 16 miles or more for 16%. By 2022, only 9% of the farthest CEs were in the same zip code, and for 51% of pharmacies it was 16 miles away or more (*P* < .001). Among pharmacies in this category, the median (IQR) distance was 35 (23-67) miles, with a 90th percentile of 176 miles.

Safety-net composition decreased over time. In 2009, 95% of pharmacies contracted exclusively with safety-net hospitals and clinics. By 2022, only 54% of pharmacies contracted exclusively with safety-net facilities, and 16% contracted with no safety-net facilities (*P* < .001).

## Discussion

We found that as the proportion of pharmacies with at least 1 contract increased, so did the number of contracts per pharmacy and the distance between contract pharmacies and CEs. However, the proportion of contracts with core safety-net practices decreased.

Study limitations include the lack of data on the volume of 340B drugs dispensed through contract pharmacies, and associated revenues; thus, we could not differentiate contracts by profitability. We also focused on retail pharmacies; thus, results may not generalize to mail-order and specialty pharmacies. Although proxies, these measures can help stakeholders better understand how the 340B program has grown among retail pharmacies.

## Notes

Supplement.
Data Sharing Statement
