# Supplementary material for: Trends in 340B Drug Pricing Program Contract Growth Among Retail Pharmacies From 2009 to 2022
Source: JAMA Health Forum. 2023 Aug 4;4(8):e232139. doi: 10.1001/jamahealthforum.2023.2139 (PMC10403775; doi:10.1001/jamahealthforum.2023.2139)
Supplement: Supplement. — Data Sharing Statement [file jamahealthforum-e232139-s001.pdf]

Data Sharing Statement

Nikpay. Trends in 340B Drug Pricing Program Contract Growth Among Retail Pharmacies From 2009 to 2022. *JAMA Health Forum*. Published August 04, 2023. doi:10.1001/jamahealthforum.2023.2139

Data
Data available: No

Additional Information
Explanation for why data not available: We are not posting the data on a public-facing website at this time but will make it available upon request. As a deliverable to our funder, we are planning to eventually publish our data on a public-facing website.
